# In-print of the environment on the molecular sensitisation profile towards pollen allergens revealed by allergen micro-array

**DOI:** 10.1186/2045-7022-4-S2-P44

**Published:** 2014-03-17

**Authors:** Viktoriya Garib, Eva Wollmann, Gulnara Gjambekova, Rudolf Valenta

**Affiliations:** 1 Medical University of Vienna, Div. of Immunopathology, Dept. of Pathophysiology, Vienna, Austria; 2 Medical University of Vienna, Div. of Immunopathology, Dept. of Pathophysiology, Vienna, Austria

## Background

The green area in Tashkent-city in Uzbekistan has been re-organized during the last two decades. It covers approximately 35% of the total area and includes classical vegetation present in Central Asia such as saltwort and newly planted species such as Bermuda grass. The knowledge of the molecular sensitization profiles of allergic patients is essential for the correct treatment with allergen specific immunotherapy but has not been established for allergic patients in the Central Asian area.

## Objective

The aim of this study was to determine the IgE-sensitization profile towards pollen allergens in patients with respiratory allergy from Tashkent.

## Methods

Fifty adult patients with allergic rhinitis and/or asthma were tested using an allergen micro-array containing 112 different allergen molecules (ImmunoCAP ISAC; Thermo Scientific).

## Results

We found that the major Bermuda grass pollen allergen, Cyn d 1 and the major saltwort allergen Sal k 1 were most frequently recognized allergens. More than 42% of the patients displayed IgE reactivity to Cyn d 1 and the percentage was even higher in the group suffering from allergic asthma (n=12) (i.e., 66%). Sal k 1 was recognized by 46% of the patients and by 50% of the patients with asthma. The other pollen allergens were recognized less frequently. Interestingly, more than 20% of patients showed IgE cross-reactivity with profilins from grass pollen, weed pollen and birch pollen whereas no patient mounted IgE reactivity to the major birch pollen allergen, Bet v 1.

## Conclusion

The results of the IgE profiling identify grass pollen and in particular Bermuda grass and salkwort as the most important pollen allergen sources in Tashkent. Although approximately 20% of the patients reacted with birch pollen profilin, none of the patients reacted with the major birch pollen allergen, Bet v 1, indicating that these patients had no genuine sensitization to birch. These data show that the molecular sensitization profile towards pollen allergens in Central Asia is an in-print of the local flora and indicates how important allergen micro-array analysis is for the selection of the correct immunotherapy treatment. This study was supported by the Austrian Science Fund (FWF), American Austrian Foundation (AAF) and was performed in the framework of International Network University for Molecular Allergology and Immunology.

